# Social Determinants and Health Equity Activities: Are They Connected with the Adaptation of AI and Telehealth Services in the U.S. Hospitals?

**DOI:** 10.3390/ijerph22020294

**Published:** 2025-02-17

**Authors:** Pearl A. Pinera, Pearl C. Kim, Fye A. Pinera, Jay J. Shen

**Affiliations:** 1 Department of Healthcare Administration and Policy, School of Public Health, University of Nevada, Las Vegas, NV 89119, USA; pinerp1@unlv.nevada.edu (P.A.P.); pearl.kim@unlv.edu (P.C.K.); pinera@unlv.nevada.edu (F.A.P.); 2 Center for Health Disparities Research, School of Public Health, University of Nevada, Las Vegas, NV 89119, USA

**Keywords:** artificial intelligence, telehealth, social determinants, health equity, hospital

## Abstract

In recent decades, technological shifts within the healthcare sector have significantly transformed healthcare management and utilization, introducing unprecedented possibilities that elevate quality of life. Organizational factors are recognized as key drivers in technology adoption, but involvement of hospitals in community-oriented activities and promotion of health equity are underexplored. This study investigated the impact of community social determinant activities and health equity activities on the adoption of AI and telehealth services within U.S. hospitals. The data were collected from the 2021 American Hospital Association (AHA) annual survey and were analyzed using multiple logistic and linear regression models to examine activities related to addressing population health, particularly social determinants and health equity, and their impacts on the adoption of AI and telehealth among U.S. hospitals. The results indicate a significant positive association between the community social determinant indicator and health equity indicator in adopting AI and telehealth services. Organizational factors were also major drivers of AI and telehealth adoption. The active incorporation of hospital strategies that address social determinants and promote health equity leads to the integration of advanced technologies and improves hospital conditions, enabling more adaptability to the changing healthcare landscape, which enhances healthcare services and accessibility.

## 1. Introduction

The widespread acceptance of technology in the healthcare sector over the past decades has led to a paradigm shift in healthcare management and utilization, contributing to enhanced patient outcomes across a broader continuum of care. The implementation of technologies such as artificial intelligence (AI) is promptly gaining traction in many medical disciplines, functioning as clinical support systems for many healthcare providers [1]. Simultaneously, the integration of telehealth has introduced significant progress in the delivery of virtual care services, further elevating healthcare processes and overall accessibility [2]. Growing health technology adoption in healthcare highlights public concerns with the received quality of care and safety risks within U.S. hospitals [3]. These have driven healthcare organizations to embrace advanced innovative solutions that prioritize patient safety and elevate care delivery.

The development of AI in 1956 significantly boomed to support human input in various settings [4] and healthcare has been no exception. AI is a designed computer system that executes human intelligence across numerous healthcare operations and has shown incredible results in different healthcare areas including service management, logistic processes, predictive medicine, diagnostic treatment and prognosis evaluation, clinical decision-making, and medical research [5]. The utilization of AI services in many healthcare operations has improved patient safety and the received quality of care, especially with rising healthcare-associated infections, adverse drug events, and diagnostic errors [6]. Additionally, with the increasing U.S. healthcare costs, integration of AI has been seen as a potential asset to reduce healthcare spending by billions of dollars in the next few years [7]. As a result, hospitals embrace the adoption of AI to streamline healthcare processes, including mitigating healthcare expenses.

Similarly, the utilization of telehealth technologies has flaunted remarkable outcomes in healthcare, particularly during the onset of the COVID-19 pandemic [8]. While U.S. hospital admissions rose with COVID-19 patients, together with complications from cardiac events and other common health conditions [9], telehealth and remote monitoring have become significant tools for managing patient care from a distance. During this period, the majority of telehealth adoption was associated with the need to provide continuity of care for paused inpatient services and an absence of hospital spaces amid the mandatory “stay-at-home” protocols [8,10]. Regardless of the decline in telehealth utilization at the end of 2021 [11], telehealth services continue to reach many healthcare practices including managing chronic geriatrics, mental health, and other specialized areas of care [12,13,14]. Despite these innovative solutions, certain hospitals still lag in the adoption of telehealth and AI, creating barriers to healthcare service delivery and exacerbating existing health disparities [15,16]. These disparities underline the need for greater technology adoption within hospitals, ensuring equitable access to care and enhancing health outcomes among broader patient demographics.

Institutional theory argues that legitimacy in environments is crucial for organizations to exist and grow [17]. Organizations often respond to changes in their surrounding environments (e.g., geographic area, local policy, population and community, and market competition) for maintaining and strengthening their legitimacy. Therefore, changes being made, such as technology adoption among hospitals, are influenced by environmental and community factors. Telehealth utilization among U.S. hospitals is significantly affected by several state-level policies related to service reimbursements, including market dynamics and technology capacity [18]. Similarly, AI adoption among hospitals is shaped by factors such as market share, hospital characteristics, and performance outputs [19]. Adding the restrictions and barriers based on hospitals’ cultural stances and overall healthcare structure, technology implementation becomes more challenging. In particular, U.S. hospitals exhibit multifaceted organizational cultures, instead of one major cultural type, with these hospitals embodying strong multidimensional cultures, using higher quality measurements, and giving patient-centered care [20]. Additionally, the complexity of diverse networks of the U.S. healthcare system raises concerns ranging from delivery and access to the overall healthcare framework. This has led to creating disparities in the overall provision of healthcare, affecting mostly less-resourced and underserved populations.

Moreover, in response to the need for broader patient demographics in communities, many hospitals, to gain and strengthen their legitimacy, are actively involved in population health by implementing strategies that address upstream social determinants of health and the core causes of health inequity/disparities [21,22]. These approaches provide a foundation for navigating the complexities of healthcare delivery and improving health outcomes [23]. Despite the importance of these initiatives to address population health, their impact on the adoption of technologies such as AI and telehealth services remains under-examined. This underscores the importance of further investigation into how these hospital activities related to social determinants and health equity may impact or hinder the adoption of technologies in healthcare settings. According to institutional theory, we hypothesized that, for maintaining and strengthening legitimacy, hospitals that are able to do well in one area, such as addressing community social determinants needs, will also be likely to do well in other areas such as AI and technology adaptation, because they often have the mindset, resources, and experiences to do so.

Nevertheless, although numerous studies have investigated the significance of AI and telehealth technologies, there is a literature gap in exploring the relationship between social determinants efforts on the adoption of AI and other technologies in hospitals. This has led to a growing lack of acknowledgement of the importance of hospitals’ involvement in activities aimed at narrowing disparities and underlying social factors across various areas. Additionally, with the current status of the U.S. healthcare system framework that continues to stir conflicts related to equitable access and delivery of care, the integration of new technologies across larger diverse demographics has become more challenging. Therefore, examining the significance of social determinant indicator- and health equity indicator-related hospital activities is currently needed to improve hospitals’ strategies focusing on addressing population health to assess greater technological adoption across all healthcare areas. The aims of this study were to (a) examine the association between social determinants and health equity activities with regard to the adoption of AI and telehealth services; (b) evaluate the association of these activities with the utilization of AI, telehealth, and the overall volume of virtual services; and (c) identify organizational factors that might relate to these technologies’ adoption in U.S. hospitals.

## 2. Materials and Methods

### 2.1. Data Source

This study chose various types of hospitals across the United States, considering ownership, market characteristics, bed size, geographic locations, and hospital groups. The aspects of hospital community social determinant indicator and health equity indicator were stated as the independent variables (manipulated variable), while the AI and telehealth services were articulated as the dependent variables (observed variable). Data for this study were obtained using the 2021 American Hospital Association (AHA) annual survey, which represents various types of hospitals, healthcare networks, patients, and communities [24]. The AHA annual survey is a valuable resource for identifying AI services, telehealth, remote patient monitoring, and behavioral or social determinants of health services [25]. The year 2021 was chosen for the period of this study as it was the only year that information on AI-related activities was available, and it also provided the latest data on health equity activities and other initiatives demonstrating equity efforts within U.S. hospitals. The final sample size included 4061 hospitals.

### 2.2. Dependent Variables

Five dependent variables were selected to illustrate whether hospitals adopted AI or telehealth and to assess the extent of use of technologies, such as AI, telehealth, and virtual services in hospital operations. The use of AI and telehealth were treated as dichotomous variables to demonstrate a hospital’s status regarding the adoption of these technological tools. The dependent variable of using AI was sourced using the AHA questionnaire, which asked whether a hospital uses AI or machine learning in any of the activities (predicting staffing needs, predicting patient demand, staff scheduling, automating routine tasks, and optimizing administrative and clinical workflows). The dependent variable of using telehealth was sourced using the AHA questionnaire, which asked hospitals to check whether it offers any different types of telehealth services (e.g., consultation and office visits, eICU, stroke care, psychiatric and addiction treatment, remote patient monitoring, other virtual care). These two variables referred to the condition of hospitals integrating AI or telehealth into their services and practices. Other dependent variables included the number of AI activities, the number of telehealth services, and the overall number of virtual services which were cited as continuous variables. The number of AI activities highlights the total records of AI appearance with regard to hospital operations. On the other hand, the number of telehealth services refers to the overall number of remote services delivered via digital health platforms. Finally, the overall number of virtual services incorporates all of the services offered virtually, including AI and telehealth.

### 2.3. Independent Variables

Independent variables were selected to indicate hospitals’ activities related to social determinants and health equity. To measure hospitals’ involvement in addressing social determinants of health and promoting health equity, we assessed two key indicators: the social determinant indicator and the health equity indicator. All independent variables were obtained using the 2021 AHA annual survey, which records all necessary comprehensive information of U.S. hospitals’ activities.

The two independent variables were composite indicator of social determinant activities and composite indicator of health equity activities, based on Section F of the AHA annual survey. According to the AHA survey questionnaire design, the first independent variable, the community social determinant composite indicator focused on addressing social determinants that are non-medical factors that influence the health of individuals. AHA has recognized different social determinants of health that implicate healthcare access, including economic stability, neighborhood and built environments, education, social and community context, biology, and health behavior [26]. This social determinant indicator variable exhibited 28 community social determinant-related programs/activities (e.g., housing, food security, transportation, interpersonal violence, employment, education) reported by U.S. hospitals in the AHA 2021 annual survey, ranging from 0 to 28, illustrating the various community social determinant activities that hospitals are involved in. The second independent variable, the health equity composite indicator, focused on accountability and commitment of hospitals in assessing the hospital’s strategies towards health equity goals such as addressing health disparities, equitable access to care, diverse representation, and culturally appropriate patient care. This health equity indicator encompassed 24 health equity actions/activities (e.g., having a health equity strategic plan, implementing and meeting health equity goals, and using DEI (diversity, equity, and inclusion) in decision-making), ranging from 0 to 24, highlighting the extent of hospital engagement in activities that promote equitable access to care and diversity. These activities primarily focused on managing health disparities and inequalities, aiming to achieve equitable health outcomes.

### 2.4. Statistical Analysis

Multiple logistic regression was used for analyzing the two dichotomous dependent variables and multiple linear regression was used for analyzing the three continuous dependent variables. Covariates being considered in the multivariable models included the hospital’s bed size (6–49 beds, 50–199 beds, 200–399 beds, and 400 beds or more), ownership (public, investor-owned, and non-for-profit private), type (specialty hospital, children’s hospital, mental health hospital, long-term care hospital, acute short-term hospital, and rural and non-rural hospital), and local market competition (competitive market, mild-concentrated market, and concentrated market). In response to the right tail distributions of both the number of AI services and the number of telehealth services, sensitivity analysis was conducted by taking the log of the two continuous dependent variables, respectively, and rerunning the regression models, which yielded similar results in terms of statistical significance of the independent variables.

## 3. Results

Table 1 illustrates the trends and characteristics of AI and telehealth adoption in U.S. hospitals including social determinants and health equity activities from 2021. AI adoption was observed at about 24.62%, with an average of 0.68 AI services and a standard deviation (SD) of 1.41. On the other hand, telehealth adoption was significantly higher at about 84.54%, with an average of 3.06 telehealth services and an SD of 2.71. The average number of all virtual services was 65,553, with an SD of 304,790. In terms of hospital activities, the mean number of social determinant activities was about 13.26, with an SD of 8.59 while health equity was about 8.66, with an SD of 8.01. Furthermore, 36.81% of hospitals had a bed size of 50–199 beds and approximately 80.06% of hospitals were short-term acute general hospitals. Additionally, the majority of hospitals were not-for-profit (60.72%, with (39.94%) identified as teaching hospitals, located mainly in rural areas (37.36%), and structured as concentrated markets (64.57%)).

Table 2 shows the associations between social determinants and health equity activities of hospitals and their adoption of AI and telehealth. A one-point increase in the community social determinant indicator was associated with a 7.0% increase in the odds of adopting AI (odds ratio [OR] = 1.070, 95% confidence interval [CI] = [1.054, 1.086]) and a 7.7% increase in the odds of adopting telehealth services (OR = 1.077, CI = [1.060, 1.093]). Similarly, a one-point increase in the health equity indicator was associated with an 8.5% increase in the odds of adopting AI (OR = 1.085, CI = [1.070, 1.1001]) and a 4.6% increase in the odds of adopting telehealth services (OR = 1.046, CI = [1.026, 1.067]). In addition, this study showed that small hospitals were less likely to adopt AI. Public and investor-owned hospitals were less likely to adopt both AI and telehealth compared to their non-for-profit counterparts. Rural hospitals had a higher likelihood of adopting telehealth and hospitals within competitive markets tended to adopt AI compared to their counterparts in concentrated markets (Table 2). The results related to organizational factors are also shown in Table 2.

Table 3 demonstrates the associations between hospitals’ community social determinants and health equity activities and their impacts on the numbers of AI and telehealth services, as well as the volume of all virtual services. A 10-point increase in the community social determinant activity indicator was associated with an increase of 0.22 in the new type of AI services and 0.52 in the new type of telehealth services, respectively. Similarly, a 10-point increase in the health equity activity indicator was associated with an increase of 0.49 in the new type of AI services and 0.75 in the new type of telehealth services, respectively. Moreover, an increase of one point in the community social determinant activity indicator was associated with an increase of 749 virtual service volume, whereas an increase of one point in the health equity activity indicator was associated with an increase of 3125 for all virtual service volume. The results related to organizational factors are also displayed in Table 3.

## 4. Discussion

The adoption trends of AI and telehealth among U.S. hospitals draw attention to the significance of hospital strategies to address social determinants and health inequalities. As hospitals engage in these population health-related activities, technology adoption increases, matching hospitals’ focus to recognize initiatives that are centered on social determinants of health and health equity, meeting population needs and demands. Our findings demonstrate that hospital indicator-related activities addressing social determinants and health equity are consistently and positively associated with the adoption and use of AI and telehealth services.

Several key factors may help to interpret this association. Primarily, hospitals’ strategic alignment with priorities centered on community initiatives and achieving equitable care served as a major driver. U.S. hospitals that respond to community health needs, as evaluated through community health assessments, improve population health and modify their healthcare services in response to community concerns [27]. For example, utilizing healthcare services for vulnerable individuals that are unique to their healthcare needs may influence hospitals to adopt technology-driven and innovative solutions to cater to a broader patient demographic. Relatedly, Carroll-Scott, A, 2017 [14] demonstrates that hospitals’ inclusion of health equity in their assessment of community health needs reflects the growing trend of population health strategies that elevate healthcare costs and enhance patient outcomes. Additionally, hospitals committed to breaking barriers through community-oriented activities contribute to improved quality care by effectively tailoring services to the needs of specific populations [28]. The rapidly changing social environment, including the increasing availability of technology, may also influence the strategies of hospitals to become more adaptive and embrace new technologies to provide preeminent healthcare services and treatment. Emerging state health policies, particularly those related to reimbursement methods, may also account for driving the adoption of telehealth and AI within hospitals [19,29]. Other possible underlying factors may include comprehensive administrative support, a well-trained healthcare workforce, and adequate hospital financial resources.

Another significant finding of our study is the positive association between hospital activities related to social determinant indicators and health equity indicators and the increased number of AI and telehealth services, as well as the volume of virtual services. In other words, the extent and abilities of providing these services may be related to hospitals’ strategic goals, particularly those focused on addressing social needs and health disparities through social determinants and health equity activities. Research highlights the significance of hospital boards’ involvement in strategic decision-making, contributing to hospitals being able to navigate the complex and evolving external healthcare environment [30]. Such involvement ensures that hospitals’ strategies are aligned with external forces and opportunities, hence enhancing the ability to respond in the rapidly changing healthcare landscape. For instance, the changing patient demographics have driven the need to improve quality healthcare and access for growing minority populations in the U.S., both racially and ethnically [31]. Shifts in disease patterns and their impacts on the population have also imposed changes in terms of healthcare delivery, technology, and health policy. Notably, the COVID-19 pandemic has created a massive impact on ramping up healthcare innovation approaches, facilitating the adoption of new collaboration methods, dynamic care strategies, and public approval of innovative solutions [32]. As healthcare continues to evolve, there is a growing focus on hospital policies that implement community engagement addressing upstream social determinants such as income, housing, insurance, and education. Hospitals’ strategies addressing non-medical factors play a huge part in improving community health outcomes [33]. Furthermore, integrating initiatives that address disparities has been shown to increase health equity, elevating the provision of equitable healthcare and accessibility. These efforts may drive hospitals to allocate specific services and develop healthcare approaches that align with population needs and preferences in advancing innovative solutions.

In addition, several organizational factors were found to be associated with the adoption of AI and telehealth in hospitals. In regard to hospital bed size, larger hospitals tend to be more likely to adopt AI and telehealth, which is consistent with the existing literature [18,19]. This is likely due to their capabilities to acquire greater technological resources and integrate innovative solutions more quickly, allowing them to adapt seamlessly to emerging healthcare trends. Hospital type was also associated with the use of AI and telehealth. Short-term acute general hospitals, in particular, tend to be more likely to adopt technologies compared to other hospitals. This may be due to the higher patient volume and the demand of various advanced care interventions, which promote the adoption of new technologies. In terms of hospital ownership, not-for-profit private hospitals are more reluctant to offer AI and telehealth services. This is likely due to their mission-driven focus and commitment to serving the community with quality patient-centered care [34]. Not-for-profit private hospitals are more likely to adopt various forms of AI than their counterparts with for-profit non-private hospitals because doing so enhances their capacity to deliver quality patient care that reflects their mission and goals [35]. Not-for-profit hospitals being tax-exempt solidifies their status and benefits them by including access concepts to their mission statement and quality services, whereas for-profit non-private hospitals exclude cost-effectiveness into their mission which strives to prioritize revenue and investors’ returns [36], which might hinder AI adoption. Therefore, for-profit ownership is more likely to be in a less non-adoption category when it comes to AI. Similarly, not-for-profit private hospitals are also more likely to implement telehealth services than their for-profit counterparts, which is consistent with the existing literature [37]. Additionally, not-for-profit private hospitals expand operations to reach a broader patient demographic, allowing individuals wider access to more holistic healthcare services. Rural hospitals are also more motivated to offer telehealth services to provide complex medical care and to enable remote specialist consultations for distant patients who face barriers to accessing healthcare [37]. Particularly, telehealth allocates rural hospitals with a value-driven approach to care for patients in remote areas. In contrast, they may not be able to adopt AI quickly due to their limited resources and market demand. Finally, competition may stir market dynamics that force hospitals to adopt recent AI technologies. In more competitive markets, hospitals are urged to adopt the latest technologies to retain their market advantage. Competitive hospitals commonly implement AI to enhance clinical outcomes, increase revenue streams, and maintain their competitive position [38]. Furthermore, competitive tensions drive hospitals to acquire technologies that attract and retain more patients and better address the unique needs of a broader patient demographic.

As hospitals move forward to address population health and reduce disparities, strategies focused on targeting community social needs in combination with health equity activities may need to be incorporated into hospitals’ strategic goals. The findings of this study are relevant for significant policy implications regarding healthcare management and the adoption of emerging technologies such as AI and telehealth in U.S hospitals. For instance, the allocation of financial support and resources to hospitals that engage in these population-focused activities can encourage healthcare organizations and other health institutions to incentivize greater technology adoption to address the barriers faced by marginalized patients. Additionally, policies that support value-based payment systems, such as the merit-based incentive payment system, can provide financial incentives for hospitals that prioritize improved care processes and patient engagement in care. Therefore, policy makers should recognize the importance of hospitals’ involvement in activities related to addressing the social determinants of health and promoting health equity in driving technology adoption. Hospitals that adopt technologies to enhance care for broader patient demographics could encourage greater reimbursement rates, allowing hospitals continued focus on activities that address the population [28]. These engagements can collectively promote the adoption of technology on a larger scale.

## 5. Study Limitations

The limitations of this study underscore the importance of further research in several areas. First, this study utilizes cross-sectional data from 2021, restricting the generalization of hospital’s related activities that address population health and the adoption of AI and telehealth in other years. Although associations were observed based on the reliance on a cross-sectional study, it may be insufficient to fully determine the order of events that occurred over time, and a longitudinal study would be needed to track these changes in events. Second, this study only relies on data from the AHA 2021 survey, which could overlook other significant key factors impacting AI and telehealth adoption. As the data were only retrieved from a single year, some hospitals might not have reported all activities related to the social determinants of health/health equity and technology implementations, leading to an incomplete understanding of hospitals’ engagement in both areas. Third, some hospitals have limited capabilities to implement AI services, creating barriers to respond to AI-related questions, resulting in missing data and complicating the measure of AI services within hospitals. Fourth, the number of AI services offered by hospitals was restricted to just five related measures, which hindered comprehensive assessment of the AI services available within hospitals. More extensive information on AI services may need to be obtained to better evaluate hospitals’ activities related to addressing population health. Another limitation of this study is the absence of a focus on commercial insurance within the U.S. system, which results in a lack of understanding of social equity in terms of patient delivery. Future research is merited to investigate the role of commercial insurance in addressing the healthcare needs of the underserved population. Finally, although this study highlights the importance of hospitals’ activities related to addressing social determinants and health equity, it does not comply with other organizational factors, particularly external forces that might drive technological adoption. Future research should take into account a broader scope of technological drivers.

## 6. Conclusions

In conclusion, this study presents evidence that hospitals that actively engage in activities that address social determinants and promote health equity are positively associated with the adoption and utilization of AI and telehealth services. Increased technology adoption within these hospitals informs the significance of strategies addressing non-clinical factors that affect health and an advancement in equitable inclusive patient care, striving to improve overall healthcare delivery. By engaging in community activities that address the social determinants of health and health equity, hospitals can enhance their capabilities to improve care delivery and access and reduce disparities regarding barriers stopping individuals from attaining the highest levels of care. This motivates hospitals to prioritize improved care processes and patient engagement through a broader continuum of care. Therefore, hospitals can enhance their capacity to adopt emerging technologies and be more adaptive as a healthcare organization. The findings also support the need to address specific healthcare challenges, such as rural healthcare access, that often hinder underserved populations from obtaining timely medical attention and the broader impact of technology adoption within hospitals. Within the structure of the U.S. healthcare system, technology implementation is significant in many areas and addressing population health and reducing disparities are two of the many aspects that mean that effective widespread technology adoption is crucial among healthcare organizations.

## Figures and Tables

**Table 1:** Characteristics of AI and telehealth adoption in U.S. hospitals, 2021 (n = 4061).

Variables	Percent
Response Variable	
Use of AI	24.62%
Number of AI services, mean (SD)	0.68 (1.41)
Use of telehealth	84.54%
Number of telehealth services, mean (SD)	3.06 (2.71)
All virtual services, mean, (SD), (n = 2893)	65,553 (304,790)
Main Independent Variable	
Community social determinant score, mean (SD)	13.26 (8.59)
Health equity score, mean (SD)	8.66 (8.01)
Hospital Characteristics	
Hospital Size	
6–49 beds	35.16%
50–199 beds	36.81%
200–399 beds	17.36%
400–500 and more beds	10.66%
Hospital Type	
Mental health hospital	6.55%
Children’s hospital	2.27%
Specialty hospital	7.57%
Long-term care hospital	3.55%
Short-term acute general hospital	80.06%
Hospital Ownership Type	
Investor-owned	18.96%
Not-for-profit	60.72%
Public	20.32%
Teaching hospital	39.94%
Rural hospital	37.36%
Hospital Market Type	
Competitive market	19.16%
Mild concentrated market	16.28%
Concentrated market	64.57%

Source: American Hospital Association, 2021; Note: AI = artificial intelligence; SD = standard deviation.

**Table 2:** Organizational factors and adoption of AI and telehealth in U.S. hospitals (n = 4061).

	AI			Telehealth		
Variables	OR	95% Cl	*p*-Value	OR	95% Cl	*p*-Value
Main Independent Variables						
Community Social Determinants	1.07	[1.05, 1.09]	<0.0001	1.08	[1.06, 1.09]	<0.0001
Health Equity	1.09	[1.07, 1.10]	<0.0001	1.05	[1.03, 1.07]	<0.0001
Hospital Characteristics						
Hospital Size						
≥ 400 beds (reference)						
6–49 beds	0.59	[0.44, 0.79]	0.0368	0.22	[0.12, 0.41]	<0.0001
50–199 beds	0.59	[0.45, 0.78]	0.0144	0.35	[0.20, 0.64]	0.1214
200–399 beds	0.70	[0.53, 0.93]	0.9596	0.38	[0.20, 0.70]	0.4483
Hospital Group						
General hospital (reference)						
Mental health hospital	0.60	[0.36, 0.98]	0.1276	0.53	[0.39, 0.72]	0.6532
Children’s hospital	0.92	[0.54, 1.57]	0.6587	0.70	[0.38, 1.30]	0.1782
Specialty hospital	0.91	[0.60, 1.37]	0.5944	0.36	[0.26, 0.48]	0.0085
Long-term care hospital	0.78	[0.41, 1.46]	0.8116	0.23	[0.16, 0.35]	<0.0001
Hospital Ownership						
Not-for-profit (reference)						
Investor-owned	0.40	[0.30, 0.52]	0.0010	0.45	[0.34, 0.58]	0.0288
Public	0.43	[0.31, 0.60]	0.0281	0.34	[0.26, 0.44]	<0.0001
Rural Hospital	1.01	[0.83, 1.22]	0.9580	2.52	[1.95, 3.25]	<0.0001
Market Competition						
Concentrated (reference)						
Competitive	1.64	[1.30, 2.06]	0.0046	0.90	[0.69, 1.16]	0.5874
Mild concentrated	1.41	[1.11, 1.79]	0.4046	0.93	[0.70, 1.23]	0.8832

Source: American Hospital Association, 2021; Note: OR = odds ratio; CI = 95% confidence interval; AI = artificial intelligence.

**Table 3:** Equity-focused hospital community activities associated with utilization of AI and telehealth and volume of all virtual services in U.S. hospitals, 2021.

Variables	Coefficient	SE	*p*-Value
Number of AI Services (n = 4061)			
Main Independent Variables			
Community Social Determinants	0.022	0.003	<0.0001
Health Equity	0.049	0.003	<0.0001
Hospital Characteristics			
Hospital Size	0.117	0.028	<0.0001
Hospital Group			
Short-term acute general hospital (reference)			
Mental hospital	−0.208	0.095	0.0293
Children’s hospital	0.006	0.147	0.9684
Special hospital	−0.157	0.086	0.0689
Long-term hospital	−0.119	0.115	0.3020
Teaching Hospital	−0.138	0.052	0.0083
Hospital Location			
Non-rural hospital (reference)			
Rural hospital	0.038	0.046	0.4013
Hospital Ownership			
Non-for-profit (reference)			
Investor-owned	−0.198	0.064	0.0019
Public	−0.305	0.056	<0.0001
Market Competition			
Concentrated (reference)			
Competitive	0.184	0.055	0.0008
Mild concentrated	0.118	0.058	0.0421
Number of Telehealth Services (n = 4061)			
Main Independent Variables			
Community Social Determinants	0.052	0.005	<0.0001
Health Equity	0.075	0.006	<0.0001
Hospital Characteristics			
Hospital Size	0.490	0.046	<0.0001
Hospital Group			
Mental hospital	−0.386	0.159	0.0151
Children’s hospital	−0.636	0.245	0.0095
Special hospital	−0.384	0.143	0.0073
Long-term hospital	−0.485	0.192	0.0115
Teaching Hospital	0.368	0.087	<0.0001
Rural Hospital	0.736	0.076	<0.0001
Hospital Ownership			
Investor-owned	−1.231	0.106	<0.0001
Public	−0.784	0.094	<0.0001
Market Competition			
Competitive	0.131	0.091	0.1506
Mild concentrated	0.439	0.097	<0.0001
Number of All Virtual Services (n = 2893)			
Main Independent Variables			
Community Social Determinants	749	929	0.4202
Health Equity	3125	895	0.0005
Hospital Characteristics			
Hospital Size	59,885	7646	<0.0001
Hospital Group			
Mental hospital	2859	27,686	0.9177
Children’s hospital	47,630	39,996	0.2338
Special hospital	9361	24,132	0.6981
Long-term hospital	2067	34,056	0.9516
Teaching Hospital	16,121	14,594	0.2694
Rural Hospital	10,095	12,448	0.4175
Hospital Ownership			
Investor-owned	−34,373	18,473	0.0629
Public	−13,454	15,341	0.3805
Market Competition			
Competitive	386	14,941	0.9794
Mild concentrated	−5065	16,212	0.7548

Source: American Hospital Association, 2021; Note: AI = artificial intelligence; SE = standard error.

## Data Availability

Data is unavailable due to the data purchasing agreement with the American Hospital Association.
